# Betaine Supplementation May Improve Heat Tolerance: Potential Mechanisms in Humans

**DOI:** 10.3390/nu12102939

**Published:** 2020-09-25

**Authors:** Brandon D. Willingham, Tristan J. Ragland, Michael J. Ormsbee

**Affiliations:** 1 Institute of Sports Sciences and Medicine, Department of Nutrition, Food, and Exercise Science, Florida State University, Tallahassee, FL 32306, USA; bdw15c@my.fsu.edu (B.D.W.); tjr16b@my.fsu.edu (T.J.R.); 2 Department of Biokinetics, Exercise and Leisure Sciences, University of KwaZulu-Natal, Westville, Durban 4041, South Africa

**Keywords:** osmolyte, heat shock proteins, gut permeability, heat stress, thermoregulation

## Abstract

Betaine has been demonstrated to increase tolerance to hypertonic and thermal stressors. At the cellular level, intracellular betaine functions similar to molecular chaperones, thereby reducing the need for inducible heat shock protein expression. In addition to stabilizing protein conformations, betaine has been demonstrated to reduce oxidative damage. For the enterocyte, during periods of reduced perfusion as well as greater oxidative, thermal, and hypertonic stress (i.e., prolonged exercise in hot-humid conditions), betaine results in greater villi length and evidence for greater membrane integrity. Collectively, this reduces exercise-induced gut permeability, protecting against bacterial translocation and endotoxemia. At the systemic level, chronic betaine intake has been shown to reduce core temperature, all-cause mortality, markers of inflammation, and change blood chemistry in several animal models when exposed to heat stress. Despite convincing research in cell culture and animal models, only one published study exists exploring betaine’s thermoregulatory function in humans. If the same premise holds true for humans, chronic betaine consumption may increase heat tolerance and provide another avenue of supplementation for those who find that heat stress is a major factor in their work, or training for exercise and sport. Yet, this remains speculative until data demonstrate such effects in humans.

## 1. Introduction and Methodology

In today’s global society, the demand for humans to perform work in the heat is increasing [1,2]. Whether affecting manual laborers, military personnel, or athletes, heat stress is responsible for approximately 620 deaths in the United States [3] and thousands globally [4,5,6] each year. As such, strategies to manage heat stress and prolong exercise or activity in the heat have been explored extensively [7,8,9,10]. Broadly, these strategies can be categorized into *physical* and *nutritional* strategies. Physical strategies may include pre-cooling, cold water immersion, misting fans, and/or altering clothing [11]. These strategies have become popular among sports medicine staff and have varying degrees of success. Alternatively, nutritional strategies, largely based upon defending plasma volume (i.e., consuming relatively large doses of electrolytes, carbohydrates, and cold fluids), also play a role in managing heat stress. Among these nutritional strategies of heat stress management includes the consumption of betaine.

The articles for this review were collected using the PubMed.gov search engine, searching the term “Betaine” alone and in conjunction with, “Heat Stress”, “Heat Shock Proteins”, “Hydration”, “Gut Permeability”, “Osmolytes”, “Performance”, and “Thermoregulation”. Searches were terminated in July of 2020.

## 2. Betaine

Trimethylglycine (betaine) is a derivative of the amino acid glycine. Betaine can be endogenously synthesized through the metabolism of choline, or exogenously consumed through dietary intake [12]. Although betaine concentrations in foods vary depending on cooking and preparation methods, grain products and vegetables such as wheat bran (1340 mg·100 g^−1^), wheat germ (1240 mg·100 g^−1^), spinach (600–645 mg·100 g^−1^), and beets (114–297 mg·100 g^−1^) are the best sources of dietary betaine [13]. Average dietary intake for Western culture typically ranges from 100 to 400 mg·day^−1^, with a mean of 208 ± 90 mg·day^−1^ and results in an average resting plasma betaine concentration of 0.02–0.07 mmol·L^−1^ [14,15]. As betaine is a short-chain, neutral, amino acid derivative, absorption across the enterocyte is thought to primarily use the sodium-dependent Amino Acid Transport System A, however sodium-independent absorption is also known to occur [12,16]. A single dose of betaine (50 mg·kg^−1^) in healthy young men (mean age: 28 years old) free of any known diseases resulted in a peak concentration of ~1 mmol·L^−1^, in ~1 h [15]. The elimination half-life of a single dose of betaine is ~14 ± 7 h, with <5% of the original dose present in 72 h [15]. However, a loading strategy of 50 mg·kg^−1^ per 12 h for 5 days in the same population resulted in a peak concentration of ~1.5 mmol·L^−1^, ~1 h after ingestion [15]. Likewise, the elimination half-life of the five-day loading of betaine is ~41 ± 14 h, with <5% of the original dose present in 8.6 days. Thus, a five-day loading protocol of betaine increases blood concentrations 50% more than a single dose and may function nearly three times as long.

### 2.1. Methyl Donation

It is universally accepted that betaine serves two primary roles in mammalian physiology [12,17]. The first is that of a methyl donor. As the name indicates, trimethylglycine (i.e., betaine) has three methyl groups, which can serve as reagents for transmethylation reactions. If this occurs, betaine is converted into dimethylglycine, or further catabolized into sarcosine, ultimately adding to the amino acid pool as glycine [12,18]. Notably, betaine metabolism supplies methyl groups for the conversion of homocysteine to methionine [19,20], and aids in the synthesis of key metabolic proteins such as creatine [21,22] and carnitine [23], especially during periods of hypertonicity [24]. These findings have led to several lines of research that examine betaine’s role in health and disease prevention [12,19,25,26,27] as well as human performance [28,29,30,31,32].

### 2.2. Osmolyte

The second role that betaine serves is that of an osmolyte. Osmolytes are organic molecules used in the regulation of intracellular fluid concentrations and cell volume [33,34]. When presented with an external hypertonic stressor, the immediate response of mammalian cells is to decrease cell volume (i.e., fluid loss) and increase inorganic solute concentrations (i.e., electrolytes) in efforts to maintain homeostasis [33]. However, this accumulation of inorganic solutes, if severe enough, can interfere with electrical signaling and protein conformation. Therefore, in order to preserve long-term cellular function, mammalian cells seek to mitigate this problem by exchanging the potentially harmful inorganic solutes for compatible organic osmolytes, such as betaine [33,35].

Indeed, when presented with hypertonic stress, membrane-bound betaine/γ-aminobutyric acid transporter 1 (BGT1) mRNA and expression are up-regulated, leading to an increase in intracellular betaine concentration [16,34]. Interestingly, once the cells are returned to an isotonic (300 mOsm) environment, BGT1 expression remains elevated for at least 24 h [16]. This may play an important role for individuals engaging in daily exercise in hot-humid environments creating hypertonic stress. Further supporting the importance of osmolyte accumulation, Alfieri et al. cultured porcine vascular endothelial cells in a hypertonic (500 mOsm) solution with and without osmolytes (0.1 mmol betaine and myo-inositol). Cultures without osmolytes present experienced a 63% mean reduction in cell number after 56 h, however cultures with betaine and inositol experienced only a 32% decrease in cell number during the same time [16]. In a separate experiment measuring morphological changes in cell cultures with and without osmolytes, the same group observed that cultures with betaine and myo-inositol grew well and maintained proper morphology, whereas those without osmolytes experienced significant apoptosis, detaching from the plates [16]. Thus, it appears that osmolytes, such as betaine, are responsible for decreasing hypertonic stress in mammalian cells, which results in preserved functionality and increased survivability.

Simultaneously, as an osmolyte, betaine acts to retain intracellular fluid and preserve the osmotic balance during hypertonic stress. As such, this has profound impacts on bolstering membrane integrity, which becomes particularly important for enterocytes undergoing heat stress (see the *Gut and Immune Health* section, below).

## 3. Heat Stress and Heat Shock Proteins

Training in hot-humid environments can result in several physiological changes. Most notably, during active heat stress, increases in core temperature lead to elevated peripheral blood flow and an increase in skin temperature [36,37]. If core temperature continues to increase, cholinergic eccrine sweat glands become stimulated and present an electrolyte solution of sweat to the surface of the skin [38]. Sweat is generated initially from the extracellular compartment, but cells will attempt to defend plasma volume and osmolality by actively excreting water to counteract the osmotic stress. Therefore, the intracellular compartment is presented with not only a thermal stress, but additionally an osmotic stress. Proteins, when presented with a great enough thermal or osmotic stress, denature and lose functionality [39,40,41].

To combat this loss in functionality, cells express a class of molecular chaperones known as heat shock proteins (HSPs), which are categorized by their molecular weight (i.e., HSP60, HSP70, HSP90, etc.) [42,43]. HSPs promote correct protein conformation by refolding denatured proteins and thereby reestablishing functionality [44]. Despite being constitutively expressed, many HSPs are considered to be stress-induced [42]. This suggests that the baseline concentrations of HSPs are adequate to maintain protein homeostasis, until additional cellular stress like that induced from exercise (i.e., hypertonic, thermal, acidic, oxidative, etc.) is applied. Interestingly, beyond the standard role of a molecular chaperone, HSPs have been reported to interact with the plasma membrane and extracellular matrix to signal immune cells [42]. This interaction may play an important role, especially in the enterocyte, with regard to the systemic immune response that occurs during heat-related injuries [45]. Of note, HSP expression is reactionary, suggesting that the response is not fully engaged until cellular damage has already begun.

However, betaine functions in a similar capacity to molecular chaperones (i.e., attenuating stress-induced protein denaturation), by stabilizing intracellular protein conformation through enhanced hydrogen bonding between aqueous proteins in the folded state [41,46]. Indeed, osmolytes have been shown to prevent or delay stress-induced denaturation as well as help refold already denatured proteins [47,48]. Further, when osmolytes are present, there is a decreased need for HSP up-regulation during times of cellular stress, indicating osmolytes are filling the role of HSPs, attenuating cellular stress [16,34,49,50].

However, a hierarchy may exist for the cellular response to stress. It has been demonstrated that thermal stress alone (42 °C in an isotonic environment for 3 h) resulted in a ~15-fold increase in HSP70 mRNA expression, but no reported change in BGT1 expression in madin-darby canine kidney (MDCK) cells [34]. However, hypertonicity alone (515 mOsm in a thermoneutral environment for 3 h) resulted in an up regulation of both HSP70 and BGT1 mRNA expression [34]. Additionally, compared to a physiological neutral (7.4 pH), an acidic environment (6.5 pH) has been shown to increase the expression of HSP72 in MDCK cells ~2-fold [51]. In the same study, when combining an acidic environment (6.5 pH) with a hypertonic insult (+50 mM NaCl, ~400 mOsm, or +150 mM NaCl, ~600 mOsm), HSP72 expression experienced a ~6-fold and ~9-fold increase, respectively [51]. Likewise, incubating MDCK cells in a medium with hyperkalemic insult (20 and 40 mM K^+^) resulted in a modest but significant ~2-fold increase in HSP72 expression [51]. Additionally, using an in vitro CYP2E1 human hepatoblastoma cell line, betaine was shown to reduce the mRNA expression of HSP70 when presented with oxidative stress [52]. Taken together, this further supports the idea that mammalian cells rely upon HSPs to defend against many types of cellular stress (i.e., thermal, hypertonic, acidic, oxidative) and that intracellular betaine accumulation may aid in this defense by acting in a similar capacity to HSPs.

Yet, organisms of higher order and complexity may not behave as is observed in cell culture models. Nevertheless, using an animal model, Dangi et al. demonstrated that goats supplemented with betaine and subjected to long-term heat stress (42 °C, 36 ± 2% RH, 6 h per day, for 16 days) produced significantly lower concentrations of HSP60, HSP70, and HSP90 compared to those without betaine [53]. Thus, similar to cell culture data, betaine was effective in combating cellular heat stress as evidenced by a reduced need for HSP expression. These data demonstrate that animals consuming betaine were able to withstand the heat stress with reduced cellular responses and could therefore, theoretically, withstand a greater heat load before experiencing detrimental consequences. In human models, Walsh et al. demonstrated that treadmill running (70% VO_2_ peak for 60 min) at room temperature (20 °C, <40% RH) was a sufficient stimulus to increase serum HSP70 expression [54]. However, to our knowledge, there is no research in humans examining the relationship between osmolyte supplementation and HSP expression. More research is needed to further elucidate these findings and to determine if they translate to human models.

**Figure 1:**
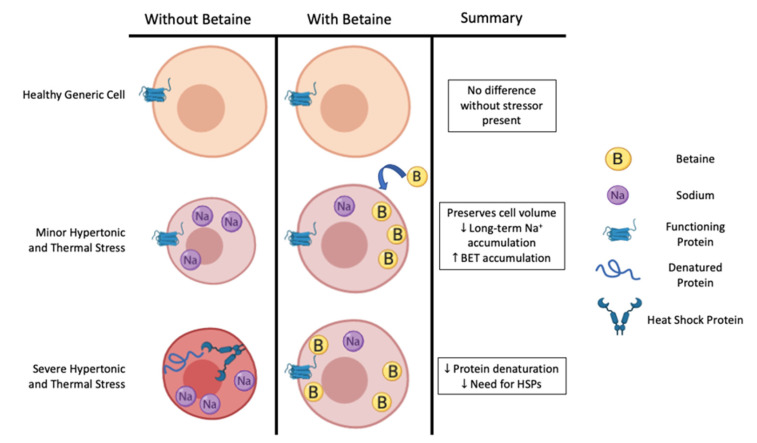
Betaine’s potential role in preserving cell function during increasing severities of hypertonic and thermal stress, as evidenced by data from cell culture models. BET: betaine, HSPs: heat shock proteins, Na^+^: sodium.

Thus, it appears that in cell culture and animal models, HSP expression is quite sensitive to many types of cellular stress that may compromise intracellular proteins’ shape and function (i.e., hyperthermia, hypertonicity, acidity, hyperkalemia, and oxidative stress). When present in great enough quantity, intracellular osmolytes appear to be the preferred method of combatting many types of cellular stress, insomuch as they attenuate the stressor and thereby delay the onset of HSP expression (Figure 1). Although not measured specifically during every type of cellular stress, it appears that hypertonicity is the specific stimulus required to up-regulate the betaine transporter [34]. Against the two primary stressors associated with exercising in hot-humid conditions (thermal and hypertonic), betaine attenuated the mRNA expression for HSP70 by >50%, suggesting a greater protection against these types of cellular stress [34]. Pre-loading betaine prior to the cellular stress may be important, as it appears that betaine is more effective at stabilizing and preventing the initial denaturation process of proteins compared to the refolding of proteins [55]. Further, there may be an additive effect if the cellular stress is too great for either method (HSP expression or pre-loading betaine) alone. If true at a systemic level, this should lead to prolonged cellular function and exercise capacity in humans.

## 4. Gut and Immune Health

Lipopolysaccharide (LPS) is an endotoxin associated with the cell membrane of Gram-negative bacteria, and is commonly found in the gastrointestinal tract of mammals [56,57]. The presentation of toxins (e.g., LPS) and gut bacteria into the blood is termed endotoxemia and is thought to be a primary pathway of heat stroke. Indeed, several studies displayed symptoms of endotoxemia in animals [58,59] and humans with heat-related injuries [58,60,61].

The mechanism by which LPS is thought to enter systemic circulation is through a breakdown of tight junctions and membrane integrity in the gut, thereby increasing intestinal permeability. As exercise intensity and the need to dissipate body heat increases, functional sympatholysis shunts blood flow away from the viscera and toward the active tissue and periphery. Physical activity has been shown to reduce intestinal blood flow by 40–80% of resting values [62,63,64,65], with even greater reductions in blood flow occurring when heat stress accompanies exercise [58,64,66]. This significant decrease in intestinal perfusion leaves enterocytes vulnerable to severe stress (i.e., hypoxia-related oxidative stress, ATP depletion, and acidosis), which results in quantifiable injury and increased permeability of the small intestine [45,67,68,69,70]. Increased intestinal permeability allows LPS and related bacteria to translocate across the tight junctions or through enterocytes into the blood [58,71,72,73]. While not uncommon for small amounts of LPS to enter portal circulation, it is thought to be quickly detoxified and cleared via the liver and never enter systemic circulation [58]. However, stressors (e.g., exercise in the heat) can lead to increased concentrations of LPS entering portal circulation, thereby overwhelming hepatic defenses and promoting immune responses and signs of exertional heat stroke [65,68].

Indeed, when severe enough, LPS in the blood causes elevated presentation of liver enzymes alanine aminotransferase (ALT) and aspartate aminotransferase (AST) in the serum. Despite these enzymes being non-specific (that is, they are known to be elevated from several stimuli), elevations may indicate the presence of hepatic injury [65,74,75]. Demonstrating this, injections of LPS (5 mg·kg^−1^) into the tail vein of rats significantly increased serum expression of ALT (LPS: 805.8 ± 245.0 units·mL^−1^ vs. CON: 38.1 ± 3.8 units·mL^−1^; *p* < 0.05) and AST (LPS: 651.0 ± 101.6 units·mL^−1^ vs. CON: 82.1 ± 3.7 units·mL^−1^; *p* < 0.05) [75]. Further, LPS in systemic circulation results in a rise in several markers of inflammation [45,70,76,77,78], most notably tumor necrosis factor-α (TNF-α) and interleukin-6 (IL-6) [74,79]. In the same study, injections of 5 mg·kg^−1^ LPS into the tail vein of rats significantly increased serum expression of inflammatory cytokine TNF-α (LPS: 20.9 ± 0.7 ng·mL^−1^ vs. CON: <0.01 ng·mL^−1^; *p* < 0.05) [75]. Similarly, 90 min after injection with 0.3 ng *E. coli* LPS·kg^−1^, humans display a significant increase in plasma TNF-α (~19-fold, *p* < 0.05) and IL-6 (~17-fold, *p* < 0.05), compared to baseline values [79]. Large increases in inflammatory markers, such as the ones evident in this study, can be suggestive of increased gut permeability and heat-related injury as temperature-dependent endotoxemia is known to result in large increases in inflammatory markers [45,69,71].

Importantly, betaine may be able to attenuate or prevent symptoms of heat-related injuries at key steps along the proposed pathway. There is some evidence that suggests betaine is able to attenuate or entirely prevent oxidative stress [52,80]. If true within the stressed enterocyte, this should minimize exercise-induced cellular damage and may help maintain membrane integrity, thereby preventing the translocation of endotoxins across the gut. In a key study, Ganesan et al., using a rat model, tested the effects of pre-loaded betaine (250 mg·kg·day^−1^ dissolved in distilled water for 30 days) on indicators of oxidative stress with and without additional restraint stress (immobilization for 6 h·day^−1^ for 30 days) [80]. Restraint stress alone resulted in significant increases in plasma corticosterone (*p* < 0.001), alongside significant decreases in betaine concentrations within the thymus and spleen (*p* < 0.001). Coinciding with the decrease in betaine concentration in the thymus and spleen, the restraint-stressed rats experienced statistically significant decreases in antioxidant function (glutathione peroxidase activity (*p* < 0.001), glutathione-S-transferase activity (*p* < 0.001), superoxide dismutase activity (*p* < 0.001), and catalase activity (*p* < 0.001)) compared to the non-stressed control group. Conversely, rats that experienced the restraint stress and pre-loaded with betaine for 30 days had a significant increase in betaine within the thymus and spleen (*p* < 0.05). Further, they did not experience a significant decrease, but were able to maintain all antioxidant markers comparable to the non-stressed control group. Interestingly, the non-stressed group that were pre-loaded with betaine did not experience significant changes in any markers of antioxidant function compared to the control group. These results suggest that betaine supplementation alone does not augment natural antioxidant capabilities but acts as another means of defending against stress-induced oxidation.

Moreover, a recent study by Wang et al. described betaine’s effect on gut health in high-salt (4.0% NaCl in the diet)-stressed rats with and without supplemental betaine (0.0%, 0.5%, 1.0%) dissolved in the water supply for 28 days [81]. Compared to the high-salt-stressed condition without betaine, betaine (0.5% and 1.0%) significantly improved markers of gut health (intestinal villi length and the ratio of villus height to crypt depth) across the duodenum (*p* < 0.05), jejunum (*p* < 0.05), and ileum (*p* < 0.05) [81]. However, betaine did not significantly alter plasma, liver, or intestinal osmolarity (*p* > 0.05), suggesting betaine was able to bolster these cells against the hypertonic stress and maintain normal osmolarity in these tissues.

These data demonstrate that betaine may reduce oxidative stress and improve gut health, both of which may attenuate LPS translocation. However, if LPS translocation remains unabated, liver damage and elevated inflammatory responses are known to occur. Yet, even in the presence of 5 mg·kg^−1^ LPS in systemic circulation, 1% betaine supplementation added to the water supply (consumption of ~1.5 g·kg^−1^, for 14 days) resulted in an attenuation of circulating TNF-α, ALT, and AST concentrations (*p* < 0.05) using a rat model [75]. Additionally, betaine supplementation has been shown to reduce total leukocyte count in heat-stressed animal models. Leukocytes are a class of immune cells that aid in the identification or disposal of foreign cells. Non-heat-stressed broiler chickens have a normal total leukocyte count of 1.14–1.17 × 10^4^ cells·µL^−1^ [82] and this value rises when presented with cellular stress [83]. Khattak et al. measured markers of immune response in broiler chickens supplemented with or without betaine when exposed to cyclical heat stress (10 h, 32–35 °C, 75–85% RH). The control group experienced a significant increase in total leukocyte count, whereas betaine supplementation was able to attenuate this immune response (BET: 1.5 × 10^4^ cells·µL^−1^ vs. CON: 3.2 × 10^4^ cells·µL^−1^; *p* < 0.05) [84].

**Figure 2:**
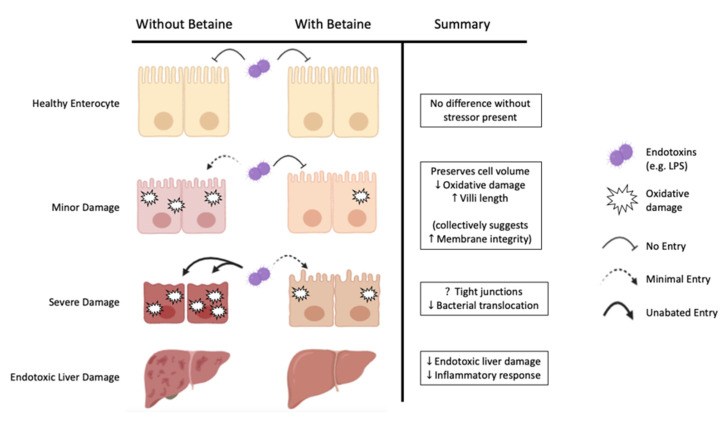
Betaine’s potential role in minimizing gut permeability through the preservation of enterocyte integrity and function during increasing severities of damage, as evidenced by data from cell culture and animal models.

Collectively, these data from cell culture and animal models demonstrate that betaine can independently reduce measures of oxidative damage, improve enterocyte health, as well as attenuate markers of potential liver damage and inflammatory responses to LPS endotoxemia (Figure 2). Thus, betaine bolsters cells against several key points in the proposed mechanistic pathway of LPS endotoxemia and may prevent heat-related injuries.

## 5. Animal Models of Heat Stress

As core temperature is a key indicator of exercise-associated GI disturbances [77,78,85,86], if betaine can attenuate the rise in core temperature during exercise in the heat, endotoxemia and thereby heat-related injuries may be preventable. The majority of this work has been performed in animal models (i.e., chickens, cows, ducks, goats, poultry, and sheep) with most demonstrating betaine to effectively combat thermal stress [84,87,88,89].

Pre-loading betaine has been shown to effectively reduce core temperature in the short (6 days), medium (21 days), and long term (63 days) in animal models exposed to cyclical heat stress. Specifically, Zulkifli et al. showed that 50 g·kg^−1^ betaine supplementation in water reduced rectal temperature (BET: 44.75 ± 0.21 °C vs. CON: 45.61 ± 0.28 °C; *p* < 0.05) in broiler chickens exposed to cyclical heat stress (4 h, 36 ± 1 °C, 75% RH) for six days [90]. Likewise, using sheep exposed to cyclical heat stress (43 °C, 49% RH, 8 h/day, 21 days), DiGiacomo et al. demonstrated that BET supplementation (2 g·day^−1^ BET in the feed) decreased core (BET: 39.6 °C vs. CON: 40.1 °C; *p* < 0.001) and skin (BET: 38.0 °C vs. CON: 39.3 °C; *p* < 0.001) temperature compared to the heat-stressed control group [91]. Moreover, Attia et al. using 1.0 g·kg^−1^ BET supplementation in the feed, demonstrated a reduced core temperature during (BET: 41.9 ± 0.50 °C vs. CON: 43.2 ± 0.60 °C; *p* < 0.05) and after (BET: 40.5 ± 0.22 °C vs. CON: 41.3 ± 0.57 °C; *p* < 0.05) exposure to cyclical heat stress (38 °C, 49% RH, 6 h·day^−1^, 3 successive days·week^−1^, for nine weeks) in slow-growing chicks compared to a heat-stressed control [92]. Further, in broiler chickens exposed to natural cyclical heating patterns (10 h, 32–35 °C, 75–85% RH), betaine supplementation has been shown to decrease the mortality rate by 10-fold (BET: 3.3% vs. CON: 33%, *p* < 0.05) [84]. Coupling these data with the reduced need for HSP expression in goats exposed to heat stress [53], it becomes evident that betaine may play a significant, direct role in managing heat stress.

Additionally, betaine supplementation may play an indirect role in managing heat stress through alterations in cellular metabolism and blood chemistry. Several researchers have demonstrated that broiler chickens without betaine supplementation experienced a reduction in mitochondrial function when exposed to heat stress [87,93]. However, betaine supplementation successfully attenuated this loss, restoring function to thermoneutral levels [87]. Although the exact mechanism is unclear, mitochondrial function may be improved due to decreased osmotic stress and thereby decreased reactive oxygen species formation [80,94]. While an increase in metabolism may seem counter-intuitive to promote heat dissipation, some researchers speculate that the decreases in lean mass and an increased mortality rate associated with heat stress are due to the inability to produce enough ATP to pant (i.e., hyperventilate) effectively [95].

In addition to restoring mitochondrial function, betaine has been shown to alter blood chemistry. Indeed, increases in red blood cell count (BET: 2.75 M·µL^−1^ vs. CON: 2.45 M·µL^−1^; *p* < 0.001), hemoglobin count (BET: 18.72 g·dL^−1^ vs. CON: 15.02 g·dL^−1^; *p* = 0.005), and hematocrit percentage (BET: 38.63% vs. CON: 28.63%; *p* < 0.001) have been demonstrated in meat-type ducks supplemented with betaine [96]. The changes in blood composition resulted in an increase in the partial pressure of oxygen (BET: 55.03 mmHg vs. CON: 37.34 mmHg; *p* < 0.001)_,_ thereby increasing oxygen carrying capacity [96]. These adaptations are thought to be a prerequisite to successful aerobic performance, especially in the heat. Therefore, betaine supplementation may not only affect heat tolerance, but perhaps also exercise performance in the heat.

Additionally, betaine supplementation has been shown to alter blood electrolyte concentrations during heat stress [84,96]. It is expected that heat-stressed broiler chickens experience a decrease in blood electrolyte concentrations, compared to chickens in a thermoneutral environment [97]. Yet, data measuring betaine’s effects on electrolyte concentrations are equivocal. In broiler chickens exposed to cyclical heat stress, betaine supplementation significantly lowered serum Na^+^ (BET: 229.4 g·kg^−1^ vs. CON: 305.8 g·kg^−1^; *p* < 0.05) and K^+^ (BET: 26.6 g·kg^−1^ vs. CON: 30.0 g·kg^−1^; *p* < 0.05), but did not significantly change serum Cl^−^ compared to the control [84]. Conversely, Park and Kim et al. using whole blood from heat-stressed ducks, found a significant increase in Na^+^ (BET: 145.52 mEq·kg^−1^ vs. CON: 126.82 mEq·kg^−1^; *p* < 0.05), K^+^ (BET: 3.17 mEq·kg^−1^ vs. CON: 2.60 mEq·kg^−1^_;_ *p* < 0.05), and Cl^-^ (BET: 120.53 mEq·kg^−1^ vs. CON: 105.61 mEq·kg^−1^; *p* < 0.05) concentrations with betaine supplementation [96]. The mechanisms by which betaine supplementation impacts electrolyte concentrations during heat stress remains unclear.

It is clear, however, that betaine supplementation has decreased core temperature, decreased mortality rate, and altered the composition of blood through direct and indirect means in several animal models when experiencing heat stress (Table 1). It is important to note that these results are in animals experiencing passive heat stress and may not accurately reflect humans undergoing active heat stress. Yet, if these data translate to human models well, it is reasonable to expect that many people (e.g., athletes, factory or field workers, military personnel) undergoing work in hot-humid conditions will benefit. More research is needed to determine if the same physiological changes occur when experiencing active vs. passive heat loads in human models.

## 6. Human Models of Heat Stress

Despite these findings in animals, there is a gap in the knowledge regarding betaine’s ability to attenuate thermal stress in humans. In fact, only one study has specifically examined this phenomenon in humans [98]. The study examined acute intake of betaine on thermoregulation and rehydration after a dehydrating protocol (2.7% body mass loss) in the heat (31.1 ± 0.7 °C, 34.7 ± 5.5% RH) in 10 male runners (age: 20 ± 2 years, height: 177 ± 6 cm, weight: 70.6 ± 6.8 kg, body fat: 6.2 ± 2.1%, VO_2_: 63.5 ± 4.1 mL·kg·min^−1^). It is no surprise that these authors report limited data supporting betaine’s ability to combat thermal stress, as rehydration after a dehydrating thermal stress effectively bypasses betaine’s preventative role in protein stabilization. Specifically, these authors found that 5 g of betaine dissolved in a carbohydrate drink did not significantly affect core or skin temperature during exercise. Yet, participants with betaine supplementation did report a lower perceived thermal sensation prior to (BET: 4.1 ± 0.2 vs. CON: 4.5 ± 0.2; *p* < 0.05) and immediately following (BET: 7.3 ± 0.2 vs. CON: 7.6 ± 0.1; *p* < 0.05) 75 min of treadmill running at 65% VO_2_ max [98]. Additionally, betaine supplementation increased oxygen consumption during the sprinting protocol (84% VO_2_ max) to exhaustion (BET: 55.0 ± 5.7 mL·kg·min^−1^ vs. CON: 52.3 ± 2.7 mL·kg·min^−1^; *p* < 0.05) [98]. Increasing oxygen consumption during exercise is universally considered to be an advantage if it translates to an increase in performance. In this particular study, although betaine significantly increased oxygen consumption, this did not lead to a significant improvement in treadmill sprint duration (BET: 228 ± 173 s vs. CON: 196 ± 119 s; *p* = 0.12). Although not statistically significant, an increase in mean sprint duration of 32 s is physiologically meaningful for athletes.

It is important to highlight that the studies demonstrating improved heat tolerance in animal models supplemented with betaine chronically in the feed or the water supply [84,87,88,89,91,99,100]. This strategy provides a daily dose of betaine, thereby loading the compound within the body. However, the human study examining heat tolerance never implemented a loading strategy but examined an acute dosage. Moreover, Armstrong et al. examined rehydration, thereby introducing betaine after the thermal stress was applied. This effectively negates the protein stabilization effects betaine has been shown to demonstrate [48,55].

## 7. Conclusions

Osmolytes, such as betaine, are responsible for decreasing hypertonic stress in mammalian cells, which results in preserved functionality and increased survivability. Betaine has been shown to attenuate many types of cellular stressors (i.e., hyperthermia, hypertonicity, acidity, hyperkalemia, and oxidative stress) that require the aid of molecular chaperones. Betaine can independently reduce measures of oxidative damage, improve enterocyte health, as well as attenuate markers of potential liver damage and inflammatory responses to LPS endotoxemia. Thus, betaine bolsters cells against several key points in the proposed mechanistic pathway of LPS endotoxemia and may prevent heat-related injuries.

Using a wide variety of dosing strategies in animal models, pre-loading betaine in the diet has been shown to reduce core temperature, skin temperature, mortality rate, and oxidative damage. Many elite and recreational athletes exercise for long durations in hot-humid environments and encounter many of the aforementioned stressors (i.e., hyperthermia, hypertonicity, acidity, and oxidative stress). Supplemental pre-loaded betaine may successfully combat these issues, as has been shown in animal models. If true on a systemic level, pre-loading with betaine may improve heat tolerance and provide an athlete who finds that heat stress is a major limiting factor, another avenue of protection in their training and performance. Yet, this remains speculative until data demonstrate such effects in humans.

## 8. Future Research

In light of this review, several lines of research become overtly clear. First, scholars should aim to translate the data from mammalian cell cultures of other species to humans. Specifically, future research should measure the effects of betaine on different types of cellular stressors (i.e., hypertonic, thermal, oxidative, acidic, etc.) in isolation and in combination, using specific tissues or tissue analogs (i.e., human enterocytes, hepatocytes, and skeletal muscle). Secondly, future researchers should test the impact of pre-loaded betaine on humans during passive and active heat stress, conditions where each of these cellular stressors are anticipated to be present. Specifically, future researchers should measure indications of thermal stress, hypertonic stress, and changes in metabolism. Lastly, future research should examine the impact of pre-loaded betaine on immune response during exercise in the heat to determine if the proposed pathway for endotoxin translocation can be improved upon through a series of exposures and adaptations (i.e., training the gut). All of these considerations will lead to a greater understanding of how athletes may use betaine supplementation to increase safety and performance in events where heat and humidity may pose a problem.

## Figures and Tables

**Table 1:** Animal models successfully using BET to combat passive thermal stress.

Author	Population	Supplementation	Thermal Stress	Significant Findings (Compared to Identified CON)
**Zulkifli et al., 2004**	Chickens (N = 150)	Ad libitum intake, water supplemented with 0 (CON) or 50 g·kg^−1^ BET	Cyclical heat stress (36 °C, 75% RH) for 4 h·day^−1^, 6 days	BET ↓ core temperature immediately post-heat stress
**Attia et al., 2009**	Chickens (N = 300)	Ad libitum intake, feed supplemented with 0 (CON) or 1.0 g·kg^−1^ BET	Cyclical heat stress (38 °C, 49% RH) for 6 h·day^−1^, 3 days·week^−1^, 9 weeks	BET ↓ core temperature during and after heat stress BET ↑ Hgb during and after heat stress BET ↓ blood pH during, but not after heat stress
**Khattak et al., 2012**	Chickens (N = 250)	Ad libitum intake, feed supplemented with 0 (CON) or 1.2 g·kg^−1^ BET	Natural daily cyclical heat stress (30–41 °C, 40–93% RH), 35 days	BET ↓ total leukocyte count BET ↓ mortality 10-fold
**Dangi et al., 2015**	Goats (N = 18)	Intramuscular injections of saline (CON) or saline + 0.2 g·kg^−1^ BET immediately prior to heat stress	Cyclical heat stress (42 °C, 36% RH) for 6 h·day^−1^, 16 days	BET ↓ HSP60, HSP70, and HSP90
**DiGiacomo et al., 2016**	Sheep (N = 36)	Ad libitum intake, feed supplemented with 0 (CON), 2, or 4 g BET daily in morning feed	Cyclical heat stress (43 °C, 49% RH) for 8 h·day^−1^, 21 days	BET (2 g) + Heat ↓ core and skin temperature
**Sahebi Ala et al., 2017**	Chickens (N = 1200)	BET as a replacement for 30% methionine needs according to methyl groups	Cyclical heat stress (32 °C, 40% RH) for 6 h·day^−1^, 31 days	BET + Heat ↔ mitochondrial Complex-1 function, whereas Heat alone ↓ mitochondrial Complex-1 function
**Park and Kim, 2017**	Ducks (N = 360)	Ad libitum intake, water supplemented with 0 (CON), 700, 1000, or 1300 ppm BET	Cyclical heat stress (33–43 °C, 70% RH) for 8 h·day^−1^, 20 days	All doses of BET ↑ RBC count, Hct, and Hgb ↑ PO_2_ and PCO_2_ ↓ blood pH ↑ Blood electrolyte concentrations (Na^+^, K^+^, Cl^−^)

BET: betaine, Cl^−^: chloride, CON: control group, Hct: hematocrit, Hgb: hemoglobin, HSP: heat shock protein, K^+^: potassium, Na^+^: sodium, PCO_2_: partial pressure of carbon dioxide, PO_2_: partial pressure of oxygen, ppm: parts per million, RBC: red blood cell, RH: relative humidity.
